# Prolonged incarceration and prisoners’ wellbeing: livid experiences of awaiting trial/pre-trial/remand prisoners in Nigeria

**DOI:** 10.1080/17482631.2017.1395677

**Published:** 2017-11-05

**Authors:** Charles T. Orjiakor, Dorothy I. Ugwu, John E. Eze, Leonard I. Ugwu, Peace N. Ibeagha, Desmond U. Onu

**Affiliations:** ^a^ Department of Psychology, University of Nigeria, Nsukka, Nigeria; ^b^ ThinkScope Consulting, Enugu, Nigeria; ^c^ Department of Human Kinetics and Health Education, University of Nigeria, Nsukka, Nigeria

**Keywords:** Awaiting trial, criminal justice system, incarceration, intervention, prison, Wellbeing

## Abstract

**Purpose**: Awaiting trial prisoners (ATPs) are represented in prisons globally, and may stay for long periods in detention. This group is however underrepresented in literature on incarcerated persons. We aim to explore the lived experiences of ATPs detained for prolonged years in a sub-Saharan country; examining what they make of their status and how their conditions have affected their wellbeing.

**Method**: Eight inmates awaiting trial for armed robbery and murder offences, held for between 8 years and 15 years participated in a focus group discussion. Hermeneutic phenomenology guided the interpretation of transcripts.

**Result**: ATPs recount disbelief and negative emotional experiences upon incarceration. Alienated and uncertain about their status, ATPs experience intensified distressful ruminations which impact wellbeing. ATPs re-rationalized incarceration and made social comparisons which breed poor perception of self. ATPs nonetheless recounted hopefulness, made favorable comparisons; and find consolation in religious beliefs.

**Conclusion**: Prolonged years spent awaiting trial fuels a deterioration of wellbeing. Alternatives to incarceration are urgently needed for ATPs. Distressful experiences recalled by the inmates beg for the inclusiveness of ATPs in programs that promote wellbeing. The Good Lives Model holds potentials for building an inclusive framework to accommodate ATPs in prison interventions.

## Background

In most countries, awaiting trial/pre-trial/remand prisoners (henceforth referred to as ATPs) make up 10–40% of prisoners (Walmsey, 2012). The proportion is substantially higher in Africa, Latin America and South Asia, where ATPs can form the majority (70–90%: United Nations Office on Drugs & Crime, UNODC, 2013; Walmsley, 2012). ATPs are non-sentenced but may be held in prison while their alleged offence is investigated, or they may be awaiting sentencing following conviction. People in the ATP category therefore may or may not have been found guilty, but it is important to note that none of them would have received a custodial sentence. Nonetheless, ATPs, especially in the developing countries, often share in the negative experiences of imprisonment with sentenced prisoners. Though they have a distinct status, ATPs are barely seen as different from convicted persons. They are generally seen as prisoners or prison inmates; perceived to be dangerous, deserving incarceration and considered people who should be isolated from society, serving term/punishment for their wrongs (Reid, 2015; UNODC, 2013).

**Figure 1.**
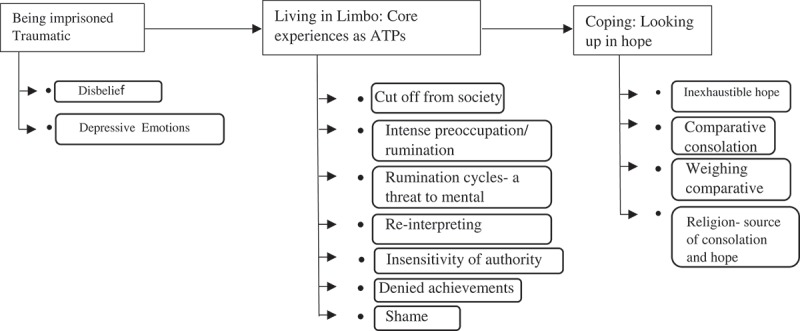
Summative presentation of themes and sub-themes.

Despite the presence of ATPs in the criminal justice systems (CJS) globally, research regarding their experiences, concerns and wellbeing is strikingly rare. In addition, most evidence on the health of prisoners comes from high-income countries (Fazel & Baillargeon, 2011) perhaps reflecting the reason why ATPs have been invisible. To better understand the experiences of ATPs, it makes sense to directly engage and discuss with people in this situation regarding their experiences. Qualitative research methods offer rich and compelling insights into the real worlds, perspectives and experiences of individuals (Braun & Clarke, 2014). Our aim here is to examine the lived experiences of ATPs who have stayed in prison for relatively long period (years) in a sub-Saharan African country, examining how their status as ATPs and their long years of incarceration have impacted their wellbeing and functioning.

One reason for the poor representation of ATPs in literature is that most offender-related studies focus on convicted offenders, as this literature is largely concerned with understanding offender/offending characteristics and risks as well as evaluating rehabilitation programs that are implemented mainly with convicted prisoners. Being convicted of an offence is usually an inclusion criterion while being non-convicted/awaiting trial/remanded is usually an exclusion criterion in most studies (e.g., see Harner, Budescu, Gillihan, Riley, & Foa, 2015). Also, ATPs include a heterogeneous mix of persons that will include people standing trial or awaiting trial for a full range of offences that may include sex, violent, and drug-related offenders. ATPs may also include political prisoners, individuals suspected of terrorism-related acts, they may also be prisoners of conscience, or illegal immigrants. Excluding this category of persons from studies to maintain the specificity of studies is understandable, purposeful, and can indeed be justified by appealing to academic rigour. However, it also means in the long term that the characteristics and experiences of numerous people passing through the criminal justice system (CJS) are overlooked and excluded in literature. Another reason could be that most studies reflecting the activities in the CJS come from developed/western countries where a majority of inmates are convicted. The outcome then is a neglected section of incarcerated persons, who, for systemic reasons may not have been convicted, but most times share similar *prisonsphere* and social climate with convicted persons. In addition, we risk not understanding how their being in prison has affected them. How are their families affected? How do they deal with the traumatic effects of incarceration? Do they share similar experiences with convicted offenders given the shared experiences of prison regimes? Do they acquire antisocial behaviours in prison making them more likely to commit offences upon release? We consider it too unwieldy to quantitatively examine these questions in one paper. However, the qualitative approach to research gifts a more liberal approach in the examination of phenomena by allowing participants to communicate their experiences without much constraints or specificity.

It is widely recognized that prison diminishes the wellbeing of individuals during and after incarceration (Fazel & Baillargeon, 2011). Close relatives especially spouses and children of the incarcerated person are also impacted (Clear, 2016; Murray & Farrignton, 2008), and so is job prospects and lifetime earnings (Clear, 2016). Severe psychiatric morbidities such as post-traumatic stress disorder (PTSD) are also reported to be common in prisoners (Harner et al., 2015). Experiences of anxiety, loneliness, powerlessness, trauma, violence, and uncertainty have been identified as general difficulties common among people in prison (Liebling & Maruna, 2005). In extension, incarceration has been identified to have spill over consequences for institutional resources in the wider community especially institutions directly responsible for health and wellbeing (Schnittker, Uggen, Shannon, & Mcelrath, 2015), putting stress on the economic, health and social safety provisions of communities with high imprisonment rates. Researchers and practitioners hence continue to stress the need to continuously probe into the daily experiences of imprisonment (Harvey, 2016). Findings from the experiences of ATPs will be vital in understanding the progress of individuals through incarceration, and can inform routine activities (e.g., assessment, formulating, implementing and evaluating interventions), geared towards better rehabilitation and overall wellbeing of inmates (Harvey, 2016). Including the experiences of ATPs will further broaden understanding on how imprisonment affects a wider range of prisoners. Therefore, in furtherance to the need for a continuous examination of experience, we commit to examining the experiences of ATPs as they are underrepresented in offender/forensic literature.

Oleski (1977) observed a high incidence of debilitating forms of anxiety among ATPs held in a detention jail in the USA. These anxieties were explained to possibly arise from the uncertain expectation of the outcome of inmates’ court hearings or prison conditions or even the welfare of one’s close relatives or dependents while incarcerated. Cassau and Goodwin (2012) found that pre-trial prisoners have a tendency to be depressed, especially during early periods of incarceration. Armiya’u, Audu, Obembe, Adole, and Umar (2013) found significant psychiatric morbidity (46%) and physical illness (18%) among both ATPs (60%) and convicted persons (40%) in a prison in central Nigeria. The latter study, however, did not specify the occurrence of physical and psychiatric morbidities peculiar to the different categories of prisoners. Specific psychiatric morbidities such as PTSD have also been reported to be significantly high in pre-trial/remand prisoners (Brinded, Simpson, Laidlaw, Fairley, & Malcolm, 2001; Teplin, Abram, & McClelland, 1996). These empirical studies suggest that ATPs share in the negative experiences of imprisonment. These studies have adopted the quantitative approach in pinpointing the presence and prevalence of health-related morbidities among ATPs, signalling their experience of distress. But the qualitative approach to research, especially phenomenology, offers an emersion in the life-world of a person/group in focus; examining experiences and meaning constructions made from daily living. The outcome is usually an in-depth idiographic account reflecting contexts and lively depictions of the range and depth of experience.

Nigeria with a population of just under 200 million people is the most populous country in Africa and the sixth most populous in the world. However, socio-political challenges have seen the country struggle to improve the lives of her people hence it is still classified a low-to-middle-income country in the over 50 years since gaining its independence from colonial Britain. The Nigerian prison system is notorious for the overwhelming number of ATPs held within it. As at March, 2017, the World Prison Brief (2017) reports that 68, 259 persons were reportedly held in 240 facilities with an official capacity of 50,153—a 125% occupancy rate, however, some reports put occupancy rates at 800%. Female prisoners make up about 2% of prisoners and another 2% are juvenile offenders; an estimated 67.9–77% of inmates in Nigerian prisons are classified as ATPs (Home Office, 2016; Institute for Criminal Policy Research, World Prisons Brief, n.d). Prison conditions are deplorable with inadequate, degrading infrastructure. Overcrowding is a common issue of concern across facilities and so is food and water shortages, inadequate medical supplies, inhumane treatments and extortions have also been reported (“Deplorable conditions in Nigerian Prisons,” 2016). Efforts by the federal and state government authorities to reduce the proportion of inmates with ATP status and improve prison conditions are reported in the media but improvements are yet to be seen. In many cases ATPs stay years beyond the 2–3 months ceiling fixed by the 1999 Nigerian Constitution, thus a breach of human rights is underscored. If a detained person finally receives court hearing and subsequently sentenced to prison, the years served awaiting trial is normally counted as time already served. Again, the Nigerian constitution provides that persons detained unlawfully be compensated and publicly apologized to upon release. However, compensations and apologies are rarely observed (Amnesty International, 2014).

With meagre resources available to prison systems in sub-Saharan Africa, ATPs’ conditions may be worse compared to convicted persons as prison systems have very limited resources budgeted for them (Penal Reform International, 2013). Like most prison facilities in the developing world, reasons such as delays in the CJS system including delays in investigation by the police, lack of cooperation by the CJS agencies, and cumbersome processes in the CJS fuel the increasing number of ATPs. Systems of this nature are also likely to be characterized by overuse of pre-trial detention making use of arbitrary arrests, a lack of access to legal counsels, corruption, etc are identified as reasons for the poor system (UNODC, 2013). Perhaps the situation in Nigeria provides a fertile ground to examine ATPs’ experience imprisonment. In this study, we probe into the daily living experiences of individuals who have been awaiting trial for relatively long years to see how they have been affected by incarceration.

## Method

### Phenomenological design

The approach adopted in exploring the experiences of prolonged incarceration by ATPs was hermeneutic phenomenology. Phenomenology is based on the assumption that a direct and subjective account of living is “knowable” (Brooks, 2015; Husserl, 1983) and therefore deals with a detailed description of the lived experiences of participants (Giorgi, 2010). Hermeneutic phenomenology reflect interpretations that showcase a co-constructed meaning integrated from the narrated accounts of lived experiences and the researcher’s theoretical and experiential repository (Norlyk & Harder, 2010; Victor & Barnard, 2016). Nonetheless, phenomenology enables investigations into areas without *a priori* answers (Giorgi, 2010). The essential phenomenological attitude of *epochē* requires the *suspension* of previous knowledge/naturalistic thinking in approaching and illuminating phenomena. The sparse literature on the experience of ATPs, particularly aids the adoption of the *epochē* outlook in exploring the life-world of ATPs. Extended knowledge will be gained from considering the experiences of ATPs who are less represented in prison studies.

### Procedure

Department of Psychology Ethical Review Board (IRB No: DPSYEC/16/03/2016B-IRB0000012) granted ethical approval and clearance for the study. The study was also approved by the prison authorities in charge of the facility. To represent the experiences of people mostly affected, we purposefully selected individuals who have spent the longest years in detention. Extreme case(s) sampling was deployed to enable us follow the focus on “prolonged incarceration” and to identify inmates deeply affected. This sampling approach allows the opportunity of selecting information-rich cases (Russell & Gregory, 2003). We decided on a criterion of ATPs who have spent a minimum of 5 years in detention. Prison officials helped to identify potential participants (n = 15) who met the criterion from prison records. Officials helped to reach out to participants in their cells, informed and invited them to participate in the study. Of the 15 identified ATPs who have spent a minimum of 5 years, 10 presented for the first meeting and were briefed on the research. Participants gave oral consent to the study and also signed consent forms. Participants were assured that any information disclosed will be treated in strict confidence by the researchers. They were also informed of their freedom to withdraw from the exercise anytime without any consequences. They completed a socio-demographic form that asked for information in areas of drug use, length of stay, accused offence, perception of guilt regarding accused offence. (highlights presented in Table I). Participants were then asked to reflect on their experiences and concerns as inmates awaiting trial and return on a scheduled date for a focus group discussion (FGD). On the scheduled date, eight male inmates reported and participated in the FGD. FGD took place during the 2-hour time given to inmates for recreation and laundry. The remaining seven inmates declined to participate. Discussion was planned to span for an hour to allow them time for their laundry and recreation.

**Table I.** Participants demographics and prison/offence profile.

Participant	Age	Length of stay	Offence accused	Substances ever used	Education Level	OccupationPre-incarceration	Previous conviction
1 Theodore	31	9	Murder	Alcohol, Cannabis & tobacco	Primary	Nil	Nil
2 Kelechi	36	13	Murder	Nil	Secondary	Nil	Nil
3 Isiaka	40	8	Armed Robbery	Tobacco	Secondary	Nil	Nil
4 Okorocha	65	15	Murder	Alcohol & Tobacco	No education	Farmer/Logger	Nil
5 Ifeanyi	31	11	Murder	Alcohol & Tobacco	Secondary	Nil	Nil
6 Sam	30	10	Murder	Tobacco, alcohol, cannabis	Secondary	Nil	Nil
7 Kalu	33	10	Murder	Alcohol, Cannabis & Tobacco	Secondary	Trader	Nil
8 Ulonna	35	14	Murder	Alcohol, Cannabis & Tobacco	Secondary	Trader	Nil

Names used in the table above are pseudonyms.

A semi-structured interview guide (see Appendix for discussion guide) was developed to inquire into the experiences of the ATPs. However, to maintain an open-ended style, an overarching comment: “*We are gathered to discuss experiences as inmates who have spent long years in prison, awaiting trial. Tell me about your experience in prison*” was used to start off the discussion. Questions in the discussion schedule probing experiences at incarceration, prolonged stay awaiting trial were used in further exploration of experience. Despite using the guide, the discussion largely flowed naturally with the moderator guiding and focusing on issues as they arise within the discussion. Discussions were recorded [this was in strict agreement with the prison authorities that recordings will only be kept and analysed within the prison]. The discussion took place in the clinic room and lasted for just over 61 minutes. Participants were walked through their experiences upon incarceration and then on their daily living in prison. As would any FGD, some individuals tended to be more engaging and willing to talk than others. However, the facilitator tried to prod quieter participants to contribute to the discussion on issues raised. Additional information was later collected in three in-depth interviews with three selected more outspoken participants. Interviews lasted 10, 5 and 8 minutes, respectively. Participants were appreciated for their time and commitment with sanitary/personal grooming effects.

### Participants

Male inmates held in a medium security prison in South-Eastern Nigeria (no female inmate was held in the facility) participated in the study. As at the time of the study, the prison with capacity of 200 inmates held 412 inmates (69 were convicted, 1 was serving a life sentence and 342 ATPs). The age of the eight participants ranged between 31 and 65 with a mean age of 37.62. Participants were recruited while the lead researcher was undergoing his clinical internship posting in a prison as a postgraduate student, and so an appreciable level of rapport existed between inmates and the intern. Prison records, corroborated by the inmates’ self-reports, identified that the participants have been held for between 8 to 15 years with a mean of 11.25 years in detention. Murder (7 inmates) and armed robbery (1 inmate) were the two offences for which participants were detained and charged. All participants, in a socio-demographic form, denied being guilty of their accused offences. There are no active formal offender rehabilitation programs in the prison, and none reported taking part in any skill acquisition program. At best, they all reported participating in Christian exhortations moderated by Christian religious groups who occasionally visit prison inmates. Prison clinic officers confirmed that none of the participants have been or is being managed for any mental illness as at the time of the study.

### Data analysis

With the authors coming from similar cultural backgrounds with the inmates/participants, the data made a richer meaning when considered in the local language (Igbo). However, it was not uncommon for participants to use a mixture of English and Igbo in communicating their experiences. Transcribed recordings were analysed in the local language and interpretations translated and back translated with the assistance of a trained linguist. Transcripts were independently read and re-read naively by two of the authors (CTO & LIU), coding and identifying meaningful themes adopting an inductive approach which demands that themes be rooted in the data (Patton, 2002). Distinct speakers were identified by their names which they were encouraged to state before making their comments. Names were later replaced with codes used on the socio-demographic forms filled by the participants such that P1 represents participant 1. These codes were later replaced with pseudonyms to vivify the integrated narrative. Transcripts with identified themes were re-analysed in a research group meeting examining and aggregating themes with the aim of piecing together the experiences of the inmates. Supporting verbatim quotes were lifted from the scripts to evidence the experiences. Where necessary and available, each identified theme was discussed in the light of pre-existing knowledge related to incarceration. Interpretation of colloquies unique to the local setting and implicit comments were explained in square brackets. A narrative description of the findings was then scripted by the first author (CTO) and edited for better illumination, tact and flow by 2 other authors (JEE and PNI).

## Findings

ATPs started off discussing their experiences upon imprisonment. Experiences of the participants reflected a mixture of disbelief, denial, and negative emotions, upon incarceration. As the discussion went deeper into daily living, inmates’ began crystallizing on their experiences in the prolonged years as ATPs. They felt alienated from society and suspended in an atmosphere of uncertainty, ATPs longed to be free and intensified thoughts on how to achieve freedom. Recognising that intensified ruminations can spiral off to a dreaded mental breakdown exacerbated psychological pressures presenting in the forms of anxious and depressive feelings. Amidst this mental pressure, inmates re- rationalized their reasons for incarceration and expressed distaste for (authorities of) the CJS, feeling powerless and oppressed. However, ATPs’ narrations were frequently encased in a hopeful outlook as inmates seem to always find a positive tilt in the social comparisons they make. Religious beliefs and convictions buffered the pressures as inmates frequently cite biblical anecdotes. Though, as expected, concern for prison conditions were reflected, discussions were mostly oriented towards coping, survival and maintaining wellbeing and a positive outlook while in prison. This is not to downplay the reality of poor prison conditions as a major source of worry, but the quest to seek out a way out of their predicament tends to overshadow the prick of the prison conditions. Indeed, an unending, *ungraduated* expectation to be free and the frustrations experienced in this endless hope, percolate to large distressful concerns and constitute a unique experience for this category of incarcerated persons. The themes uncovered in the discussion are presented below. See Figure 1 for a graphic chart of discussed themes.

### Traumatized by imprisonment

#### Disbelief

Many of the participants recalled their early reaction to being committed to prison as ATPs. They recalled being shocked, traumatized, downcast, and a struggle to come to terms with imprisonment. As all the participants reported that their current stay is their first time of being incarcerated, their accounts were roughly similar in reflecting experiences upon incarceration. Participants gave accounts that typified the shock of being imprisoned. Isiaka recalls:“…when I was brought in …I use to hear of prison, prison, I never never believed that I will one day be in it.” Also the prison condition and the older inmates encountered upon arrival formed part of their cheerless experiences. Ulonna told of his first day and how it went down with him:

When I crossed those gates, I saw men, men everywhere walking about, … then I saw the cells, I was still looking, tears dropped off my eyes, so I am now in prison and I’ll be here with these people…, I couldn’t believe it, it was hard.

The narrations of experiences upon entering the prison for the first time, portray people fixed in shock, and struggling with the reality of becoming a prisoner. Participants demonstrate that being imprisoned was not an experience they ever imagined will come to them, seemingly shattering their worldview and life expectations. Everything seems to stand still and take a sudden unexpected turn/stop for them upon stepping into the prison. The narrations of participants also showcase the shock and hard struggle to come to terms with the reality of imprisonment, and also points to the onset of emotional distress that was building up.

#### Depressive emotions

The expression of shock and disbelief gradually distilled into more lucid accounts of negative feelings. Inmates narrated the inner pain and anguish that they experience(d). Feelings of sadness, having weepy spells, losing interest in everyday living dotted their description of their earlier experiences. Theodore recalls: That time, my mind was heavy, nothing is funny, nothing was interesting, I felt very sad, very very sad… even now it was annoying.” Another respondent recalled: “when I came in newly, I was not happy at all…sometimes I fall asleep and sleep deeply… you wake up and find it is in a prison yard [hisses], you become sad. (Kelechi)

ATPs describe their experiences with tones of deep psycho-somatic experiences. They talk of *the mind being heavy* and recalled negative affective states in the early days of being incarcerated. The sadness also seems to exude frustrations and anger that seem to linger on. Cassau and Goodwin (2012) found an onset of pathological depression in the early days of incarceration among pre-trial inmates. This highlights the importance of reflecting on the nature of trauma impacting inmates as they enter the prison. There is probably a need to “inoculate” potential inmates (convicted or pre-trial) before incarceration. It is possible that the initial traumatic effect of imprisonment, if unchecked, go on to take a toll on the mental health and wellbeing of inmates.

### Living in limbo—experiences as ATPs in prolonged stay

#### Cut off from society

In addition to the negative emotions of imprisonment, inmates expressed feeling separated and alienated from general society. Words/phrases such as “outside” and “getting out” were used in referring to the general environment outside prison and being released from prison respectively. The link with the outside world was important in countering the pricks of incarceration—a link which was absent. Okorocha recalls: “There is no phone in here so that maybe when you are bored, you can talk to people on the outside to feel better….” Theodore recalls how much he feels from being pulled away from his ancestral heritage: “As I am…, I have not seen my father’s compound in 10 years now, my people just go occasionally… I don’t know how things have changed… I don’t even know if I’ll ever see it again.”

The ancestral ownership of homesteads comes through in this expression especially for natives. The words “my father’s house/compound” is usually understood in the local setting to refer to a place where one can lay unrivalled claim; one’s undisputable heritage; where a person rightly belongs and cannot be rejected. Imprisonment is therefore conceived to take away much more than freedom but is also considered as usurping one’s identity, heritage and background. This feeling of alienation in the discussion suggests a strong feeling of being cut-off from not just society but also one’s roots and was of significant concern for ATPs. ATPs also express doubts regarding their hope to get over their ordeals and reconnect to their societies as Theodore’s words highlight.

Birmingham (2003) noted that events and concerns in the outside world continues to have an effect on the life and wellbeing of incarcerated persons as they border about family, children etc. This is particularly worse for remanded prisoners as they continue to face mounting anxiety from the uncertainties in their situation.

#### Intense preoccupations and ruminations

Cut off from society and residing in prison for relatively long periods constrains the life engagements of inmates. Beyond the physical deprivations, ATPs repeatedly discuss the mental burden of their situation. They describe an unending expectation, preoccupations, and ruminations running through their minds, fostered by the uncertainty of their prison status. Perhaps this is the first somewhat unique experience reported by the ATPs and it appears to resonate in other reported experiences. Most participants described finding themselves in frequent circular ruminations, occupied with thoughts of when and how they will exit the prison. Often they report this worry as a top concern and also as constituting enormous burden on their psyche. Okorocha describes his engrossment with leaving the prison: “All I can think about is how I will get out of this place…I just keep thinking when, when will I leave here, I just want to get out.” Another participant (Ulonna) comments that: “… for me the worry is to go outside (from prison) …every other thing outside there that I am hoping for is just an added bonus.”

The burdensomeness of this preoccupation on the inmates’ psyche was often highlighted especially as they understand their length of stay to be undetermined. As no sentence has been given and sometimes little to no progress with court processes due to hindrances of funding or lack of resource persons, the situation feels even more hopeless for these ATPs. This expectation of freedom and attempts being made to secure freedom is considered a basic high-level need for the ATPs. Ogbonna considered it “*all he thinks about*.” These cycle of thoughts, often described as being recurrent by the ATPs, and to which they seem to find no resolve threaten the mental wellbeing of inmates. Kalu narrates:

“My only worry is just how I will get my feet out of this place… you know, it is heart-breaking. You wake up you start worrying, the same things…you just sit and think and think, it can make you mad.” (Kalu).

Being preoccupied with thoughts of exiting the prison were always discussed with expressions of distress hinting on the pressure such rumination put on the psychological wellbeing of the inmates as can be seen in the narration of Kalu. Birmingham (2003) identified that remanded prisoners awaiting trial face considerable uncertainty about their future. Different patterns of preoccupations have been identified in different categories of prisoners. We deduce from this segment that imprisonment may distort, or to say more conservatively, alter cognition in remarkable ways. Reflections portray a seeming pigeonholing and intensification of thought contents and processes within a limited (apparently negative) range. The uncertainty of their prison status makes the situation more daunting for the inmates sending them down a more depressive path. More terrifying for the ATPs was being confronted with the possibilities of a damaging effect of this cognitive pressure as they witness fellow inmates who break down with mental illness while being held.

#### Ruminating cycles—a threat to mental wellbeing

Beyond considering preoccupations and mental ruminations potentially damaging to mental health, ATPs further recall witnessing fellow inmates breakdown with mental illness which they link to the intense mental pressure coming from worrying. Inmates recount experiences of people who worried so much that they break down with mental distress. P9 narrates that:

…it [getting out] is always on my mind …if you don’t take it easy with thinking, you will just go mad…yes, it can make you mad…you just have to stop yourself from worrying …people who have lost their minds are many here.

The realities of mental illness resulting from the pressures of imprisonment are so lucid to inmates resulting in some kind of anxiety in inmates as demonstrated by Ifeanyi:

…they are there [pointing towards the holding area for people with serious mental distress] … they were not like this when they came in…when you think too much about what you are going through…their minds could not carry it…so their heads have gone bad…it is a fearful thing.

Psychiatric problems (of psychoses, neuroses, personality disorders etc) are reported to be proportionately higher among remanded prisoners (Birmingham, 2003; Maden, Taylor, & Brooke et al., 1995; Singleton, Meltzer, & Gatward, 1998) and these mental health needs are largely unmet. Watching other inmates breakdown with serious mental illness and linking it with the effects of imprisonment brings anxiety as well as heightens the already culminated depressive feelings intensifying the mental burden of the inmates.

#### Re-interpreting incarceration

It was interesting to see how offences were discussed in the FGD. There was a striking de-emphasis on the offences for which inmates were originally detained when raised in the discussion. In the socio-demographic questionnaire, inmates were specific about the offences for which they were detained and in addition indicated innocence regarding their charges. However, in the FGD, inmates discussed reasons for their detention in ways unrelated to offences. Incarceration for the ATPs, was viewed not in the scope of being accused offenders, rather they seem to consider themselves to be incarcerated because they are powerless; they consider themselves victims- victims of a dysfunctional, oppressive and corrupt CJS. Isiaka identified that: “I am only here because I don’t have anybody. It is unlawful to keep people here this long.” Inmates saw their prolonged detention not just as being unjust but also as an outcome of not having enough resources and “connection” to facilitate their leaving the prison. ATPs view their incarceration to be more of oppression than relating to accused offences. Ulonna reflects the frustration and feeling of not having a person to sought out his issue:

…as you see me I carry a court bail [have been granted bail], I do not know why they have still kept me here…all that is left is that I don’t have any *leg* [influential/willing person] to go and inquire what is happening… I have paid the charge and bail [lawyer], but they are just keeping me here for nothing.

Perhaps the prolonged stay for these ATPs contribute to the reason for distancing themselves from accused offences and now focus on a perceived cruelty of the CJS. Comments by Ulonna demonstrate the uncertainty and frustration experienced by inmates, concerning the difficulties in navigating and getting the right treatment from officials of the CJS. Thus ATPs appear to increasingly consider their incarceration to be unrelated to any accused offence, rather a product of a shady and oppressive system. ATPs consider themselves unfortunate and blame their misfortunes on not having “friends in high places” or well-meaning/positioned advocates to facilitate their release. Hence, in addition to the mental burden of incarceration, subtle themes of *powerlessness* and *being oppressed* can be drawn from the ATPs narratives.

We were drawn to the little concern shown by the ATPs for their accused offences. It was noticed that as the inmates became more settled in the discussion, subtle omissions and acknowledgements suggesting culpability surfaced opposing their stance in the socio-demographic questionnaire. Reacting to suggestions that people are imprisoned for some offence, Kelechi retorted “Stanley {*not his real name*} in cell B did nothing!… he was just around where the police came to make arrests and was picked up and brought here…”. It is particularly striking and worthy of note that none of the inmates raised their personal stories of innocence regarding the offences for which they were detained rather, the ATPs continued to make external references. Another reflection of experience, *comparative guilt* (to be discussed later as a coping measure) where inmates compare their offences to others perceived to be innocent, is yet another hint to a conspicuous silence by the ATPs regarding their accused offences. This reflection of experience seems to suggest that prolonged stay in detention may have a tendency to, in cases of true culpability increase denial while reducing feelings of guilt and sombreness which are important in offender behaviour change.

It is important for us to state clearly that we are not in any way suggesting that ATPs are more often than not, guilty of the crimes for which they are detained. Our interpretation may indeed be limited to the few people who were engaged in discussions and indeed the Nigerian CJS. However, in as much as we try not to adjudicate the innocence claimed by participants, we highlight that on one hand, prolonged imprisonment without trial may make inmates have an overburdening feeling of injustice and oppression which may then translate to inmates’ re-construing reasons as to why they were incarcerated.

#### Perceived insensitivity of authority figures

Inmates expressed their dissatisfaction for authority figures in the CJS. Sam puts it rhetorically “Does it concern them? …they [warders] don’t help matters at all”. Another inmate (Okorocha) simplifies the role he perceives the prison staff to be playing: “they are just here to give you food and lock you up when the time comes… you can’t even discuss your troubles with them… for them you are already condemned”. ATPs communicate that they are already viewed and treated as convicted prisoners and perhaps treated with less regard. The warders are part of the everyday lives of inmates, yet they are perceived to be cold and distant and to be of little help in addressing the concerns and ameliorating the pains of the ATPs. We note from Okorocha’s excerpt that inmates had an inclination and may actually be looking up to the prison officials for psychological/emotional support and help especially as professional psychological help may not be adequately provided in (Nigerian) prisons. Reid (2015) identifies that those who work within the CJS appear to be most desensitized about the wellbeing of prison inmates. We see here the need that ATPs equally indicate a need for psychological help and that this need can only be picked up by informed personnel. There is hence a need to pay adequate attention to the psychological health and wellbeing of inmates (convicted or awaiting trial). Prisons officials also need to be trained to be able detect these needs and maybe have some level of helping skills to assist in improving inmates’ mental wellbeing.

#### Denied achievements

Participants discussed their personal aspirations and concerns in the terms of “counting losses” and being “denied their achievements.” They were strongly concerned that their personal goals and concerns are being delayed, making them develop a sense of doubt in their ability to attain a satisfactory life. Comments portrayed feelings of being delayed and possibly denied of life’s goods. Ulonna narrates: “When I… think of what I have not achieved, if I were outside… I would have got it.” Kelechi also gives some specific desires he looked up to: “…many of us are not married, I have not built my own house, I have not driven a car, … and many of us are this way… just locked in here, when … will I even do these things?”

Ifeanyi gave a more personal relating experience:

…in these years I have been here maybe you may not have started what you are doing in school [*academic program*] that brought you here to talk to us but you see I have been here all these years counting my loss… asking myself when will I be free to do my thing.

ATPs accounts reflect not only a sense of lost time and desired life goods, but also their prolonged indefinite stay put them in a situation where they query their ability to reconnect with their worlds and follow their aspirations- raising self-doubts and compromising their self-concepts. We can deduce that their prison status impacts their self-concept especially self-efficacy and self-worth. Stretching interpretations a bit further brings to light the fittingness of the inmates’ outlook and experience to the tenets of the Good Lives Model (GLM, Ward, 2002; Ward & Stewart, 2003). The GLM posits a positive approach to the management of incarcerated persons. The model considers the pursuance of primary goods that are naturally rewarding and necessary in attaining wellbeing to be basic to humanity, imprisoned or not. Inmates communicated their desired goods of excellence in work and agency, relatedness, etc. ATPs therefore consider their prolonged imprisonment to impede their active pursuance of human goods and this feeling of being limited breed self-doubt which may affect how they see themselves fit for the larger society upon release. A punctured self-concept and feeling impeded in their pursuance of primary goods underscore a sense of humiliation experienced when they take a broader look at their situation in the broader social context—finding themselves almost always on the lower end of the social ladder.

#### Shame

Building from the negative self evaluation upon assessing denied achievements, the ATPs talked of feeling ashamed of their place in the society compared to their mates. Kalu “It is a shame to me all these years…when I get out how will I get along with my peers. I am here and my mates are climbing… it pains me so much… the shame is…big.” The shame accruing from their losses may also be linked to the stigma of imprisonment. ATPs especially as the inmates account for their losses in long years in prison as Isiaka put it: “I am in pain and in shame… if I had been pursuing my own affairs you won’t be here questioning me. So if I think about it it pains me and I feel ashamed.”

Inmates convey that imprisonment has set aback their life expectations and their shame regarding the expected societal status as portrayed in Kalu’s reference to peers showcases the loss of face. It also portrays that their status as prisoners though non-convicted is shameful for the ATPs and brings some form of pain or psyche ache.

### Coping—looking forward in hope

Though most of the experiences communicated by the ATPs were lamentations and expression of poor wellbeing, inmates reported drawing from different sources to cope with their experiences. With the broad compass of hope, inmates accepted their situations, compared themselves with other inmates, found consolation in religious beliefs as expressed in their use of biblical anecdotes, and talks of self-stamina and optimistic living to relate their experiences of surviving imprisonment without trial.

#### Inexhaustible hope

Inmates discussed their struggles to put up with their prolonged stay in prison as ATPs to be sustained by maintaining a hopeful attitude and outlook. “Here there is no hope other than the one you gave yourself.” (Isiaka). Isiaka’s recognition that meaning in their situation can only be found by the prisoner demonstrates recognition that personal struggle, perseveration and conviction towards wellbeing essentially supports them through their experiences. As inmates struggle to facilitate the judicial procedures, and face repeated frustrations, they report hanging up to hope that they will one day become free. Kelechi captures how he puts up with the daily living routine and how he handles the expectation of release:

… at a time, you just stay… worry, you think, you endure it, sometimes you cry…, when you wake up, you sometimes feel and hope that some good news will come but the day ends with nothing… but you always think how it will happen, maybe one day, a person picks your case and *piam*!, you are going… you just hope that one day something will happen and I will leave this place.

The anchor of hope seems to be the substrate upon which inmates find meaning and the strength to put up with their distress. On this hopeful outlook, all other measures of coping seem to arise.

#### Comparative consolation

Inmates compared themselves with significant others within and outside the FGD group in terms of how much years they have spent awaiting trial. Identifying individuals who have stayed longer years seem to give inmates a sort of consolation that they are not the worst of all after all. P10 demonstrates the feeling that this strategy appears to minimize distress among inmates:

…you are talking about 10 years [laughs]…it is really small for some people who were here before us. They have stayed here for 20-something years …yes [laughs] 20 something years. So when you remember that, you come back and know that there are people you are better off.

Kelechi and Okorocha’s interaction also gave a hint to comparisons:

…you think it is not true? See this papa (an elderly participant) here. How many years have you been here? [Okorocha (papa): I’ve stayed 15 years] …and some people stayed 20 [Int: without trial?] …yes, 20, it means I have not started…. [laughs].

Being through distress with others and seeing other people go through even more distress help to make the situation more bearable for the ATPs. Inmates tended to evaluate their sufferings with that of others in apparently similar or worse conditions and draw consolation from their experiences thus helping them become more resilient and have a better outlook regarding their experiences. ATPs comparison extend even to non-incarcerated individuals- considering that being free is not a guarantee to achieving satisfaction or fulfilment: “You see I have been in here for 8 years now, somebody on the outside who may be reckless or not careful may be there these 8 years, even 10 years and still not make money…” (Isiaka).

#### Weighing (comparative) guilt

This is yet another comparative position helpful in coping with the long years of imprisonment without trial. The ATPs appeared to draw relative consolation from the experiences of people they perceived as not being guilty, yet imprisoned for long periods. Inmates’ descriptions included fellow inmates and also biblical anecdotes.

There are people here when you hear their cases, you’ll laugh… they’ve done nothing! …even Joseph in the Bible, just refused… and was thrown into jail… so when you see that some people are in prison, you look at what you have done that brought you here. If you find that you have done something, then there is some justice in it and you, you take consolation… [Int: Even if you are here without trial?], You did something.” (Ifeanyi)

Taking into consideration biblical stories and the consideration of guilt in accused offences, the distress of being remanded in prison seem to whittle a bit for ATPs. In addition, ATPs talked about taking consolation on the perceived innocence of some colleagues held in the prison as well as narrating biblical anecdotes of people who were innocently imprisoned. Isiaka recalls a seeming counter to an innocence stance:

…some of the inmates here did nothing but have stayed for a long while and seeing that, you take consolation that looking at it, you know, in what you have done, you soothe yourself… some people are here and they did nothing.

As earlier stated, these (Isiaka’s & Ifeanyi’s comments) may be subtle admission of guilt in the inmates’ account. Despite the suffering and anguish of imprisonment and long stay awaiting trial, the participants find some justice in their being detained if they perceive they share some guilt in the offence for which they are being held and prosecuted.

#### Religion as a source of hope and consolation

Inmates, all identifying as Christians, made reference to their religious beliefs and activities as a source of hope and encouragement. Biblical anecdotes of the long years of agony of the “woman at the pool of Bethsaida,” (Ulonna), the long years spent in misery by the “prodigal son” (Isiaka), and the unjust “imprisonment of Joseph” (Theodore) were used by inmates to communicate and express hope and consolation at least that biblical stories also mirror their situation. “Yes, even the other place where they talked about the Prodigal son… you see after his sufferings, nobody gave him hope, he gave himself, took courage and left there.” (Isiaka)

They equally interpret their relative wellbeing and survival in prison to the belief in the presence of God.

… I will say God is with us, God is wonderful. … the condition in here is such that people should be dying maybe monthly, because we eat nothing, but you see us we look healthy, Year to year and year to year, this is the way we appear, I can only say that God keeps us that way…. (Theodore)

This steady hope was communicated in such a way that even the number of years spent in awaiting trial in prison tended to be somewhat casual in the narrations given by Theodore:

…you talk to yourself, that it shall be well, waiting for God’s time. May be you hit 5 years, you wait, 10 years, you are still waiting…you just wait for that God’s time… what else can you do? You have patience and hope….

Finding meaning and consolation in biblical anecdotes could on one hand reflect hope drawn from religious beliefs and on the other hand indicate a relative resignation of their situations to external (supernatural) factors implying that they’ve at least to some extent a diminished expectation that the CJS has the effectiveness of resolving their situation. Again the chunking of years spent in prison into bits aligns with the concepts of reduction and re-interpretation described by O’Donnell (2016) to be used by prisoners held in solitary confinement as a coping mechanism.

## Discussion

We examined the lived experiences of persons awaiting trial for prolonged periods. Findings from this study suggest that ATPs share in the pains of imprisonment, possibly in the same manner as convicted prisoners. Accounts of the inmates suggest a significant breach of human rights which corroborate reports of high abuse of human rights in low income countries (ref). Similar to findings reported on the effects of incarceration (e.g., Liebling & Maruna, 2005), reports of trauma, depression and anxiety upon imprisonment were recounted by ATPs. We received reports of stricter restrictions, poorer diets, absence of recompense following exoneration (unless prosecuted by the concerned victim) and further abuse of human rights. ATPs felt cut off from the (*outside*) society and alienated from their (cultural) identity. Living in limbo about their conviction status especially regarding the uncertainty about how long they have to stay, ATPs tend to be always on the edge (increased anxiety), often preoccupied with thoughts of exiting the prison system, resulting in a vicious mix of cycles of depressive and/or anxious ruminations. The wellbeing of inmates is further threatened by an awareness of the devastating impact of ruminations as evident in witnessing inmates who break down with mental health problems. The prolonged years without a sentence seem to make the ATPs loose connect with the offence for which they were incarcerated and consider their imprisonment a result of powerlessness, oppression, and cruelty of the CJS hence they consider their imprisonment to be unrelated to accused offences. They further develop wariness for the CJS and its insensitive agents who already consider them guilty and deserving their lot. The period spent in detention is then regarded to be lost time and the ATPs feel denied the opportunity to pursue desired primary goods. This loss put in frame of personal and societal expectations result in a denigrating impact on inmates’ sense of self. They equally experience shame when they make social comparisons.

Though all the ATPs indicated innocence regarding their accused offenses, subtle omissions and admissions on course the FGD may suggest otherwise. However, their uncertain condition underlined by feelings of powerlessness and oppression shifts more and more the issue of guilt. However, inmates look forward in hope, taking solace in diverse forms of social comparisons; comparing themselves with inmates who have stayed longer or served longer prison terms and “free” people who may not have achieved plenty by not utilising opportunities. Some may take solace in making subtle recognition of their culpability especially when they consider the pains of truly innocent figures. Religious anecdotes, beliefs and symbols were also commonly used by the inmates.

What then have we learnt about ATPs? First, this study adds to the sparse research available on ATPs. We found in the narrations of the ATPs that they experience the pains of incarceration and that their wellbeing is affected with a pronounced impact on mental wellbeing as well as their self concept. Awaiting trial for long and indefinite periods can pile up enormous mental pressure that could impair the wellbeing of individuals. Worse still they can be at the brink of developing severe psychopathology as communicated in the experiences of the respondents. Green (2017) thinks it possible, albeit an unpopular perspective, that people can *think* themselves into insanity. Also, the DSM-IV-TR (APA, 2000) recognizes “thinking too much” as a common idiom of distress in many cultures. Comparative studies are needed to see how their pains and experiences differ in magnitude from convicted prisoners—so are they possibly in more pain or distress than sentenced inmates?

Secondly, inmates’ perception of prison officials as being non-empathic, cold, hostile and insensitive to their concerns and wellbeing is for us, a red flag. Not only are these inmates impacted by the negative effects of incarceration, an indefinite incarceration with accounts of human rights abuses, the insensitivity and hostility of officials worsen the wellbeing of inmates. This is troubling especially where there is little provision for trained mental health personnel. There are studies that corroborate this hostility in similar populations. Jenkins (2013) found in a discourse study in the UK, that the prison service put immense undue pressure on inmates (who are appealing their cases) to admit guilt. Reid (2015) also identified that CJS workers close to the inmates are quite insensitive to the distressful experiences of inmates. Fisher (1972) gave a graphic description of the grumpiness, hostility, and harassment that staff (including counsellors) in a facility for children awaiting trial meted out on the inmates. Interestingly, Fisher traces the source of this hostility up to the legislative cadre.

Philip Zimbardo’s famous prison study (Haney, Banks, & Zimbardo, 1973) identified that the social climate of the prison coerces individuals to accept and play authoritarian or submissive roles. However, we are yet to comprehend why authority figures go beyond demanding absolute compliance to prison rules to accepting and treating inmates (no matter the category) as guilty. Though Zimbardo explained the prison guard-prisoner grind and persistence in roles to emanate from pressures to conform to group norms and deindividuation processes, perhaps another process is at play. Deeming ATPs already guilty may precursor prison officials’ coldness, hostility, and insensitivity to needs. Else the hostility recounted by the ATPs may also indicate that prison systems in the setting studied, are still operating (or hopefully evolving out) of the dominantly punitive (rather than rehabilitative) framework. Beyond being hostile, inmates’ discussions also suggest that they tend to look up to the guards for psychological help. This highlights the need for workers within the CJS to, at least be aware, as well as be able to recognize, and respond to the psychological needs of prisoners. Early detection of distress could improve the detection of mental difficulties and help save costs and even prevent more extreme events common in forensic settings (e.g., suicide).

Third, we identified the potentials of the qualitative approach in identifying denial. As seen in the results above, while all participants indicated innocence in the self-report styled questionnaires, the dynamics of an interactional style to data collection with focus on experience led to utterances that raise doubts in the original stance of the ATPs. None of the respondents discussed on their “personal innocence,” rather the ATPs dwelt on the perceived innocence of some incarcerated persons. Also in discussing coping experiences (e.g., *weighing comparative guilt*) inmates referred to experiencing guilt as a way of accepting their situations. Though we distance ourselves as much as possible from adjudicating their cases, we consider the dynamics of the discussion to be quite insightful and to hold potentials in navigating denial.

Lastly, it was interesting to see that amid the distress and mental burden, the ATPs indicated that maintaining a hopeful outlook gave them the pull to tarry in their uncertain situation. The ATPs habitually ended their comments on a hopeful note. This study is not alone in identifying that the discussion of inmates regarding their incarceration experience tilted towards a positive pole. Qouta, Pumanaki, and Sarraj (1997) identified from interviewing political prisoners who experienced torture in the Middle East that recounted experiences were more of positive prison experience than negative ordeals. Though this could, however, be related to the background of the prisoners and their political perception of their being held. Putting up against these conditions is suggestive of post traumatic growth.

In many ways, we see some of the coping approaches adopted by the inmates to align with the descriptions given by O’Donnell (2014, 2016) on how inmates held in solitary confinement adapted to their situations. O’Donnell examined documentations from different sources (including prison chaplain tracts and official publications) how different people over the past 2 centuries have coped with solitary confinement. We find some consonance with at least 2 of the 7 Rs (rescheduling, removal, reduction, reorientation, resistance, raptness and reinterpretation) identified by O’Donnell in the experiences communicated by the participants in this study. The chunking of years spent in prison into bits reflects rescheduling- using different intervals to estimate the passage of time. Reinterpretation in O’Donnell’s view stands for the reframing of prison experience so that its meaning and significance is rooted in more acceptable/rewarding anchors of say religion or political opinions. In the case of inmates awaiting trial, they re-interpreted their conditions to result from a cruel CJS and they also found succour in religious references which they perceive to approximate their experiences.

### Implications for practitioners

Global prison records recognize that nearly all countries of the globe have a significant proportion of prisoners who await trial and that in some cases their stay in prisons are prolonged especially in developing regions. This category of prisoners is usually excluded from offender rehabilitation/intervention programs for *rational* reasons. Nonetheless, the findings of this study suggest that the pains of imprisonment and its impact on psychological wellbeing are equally experienced by ATPs and hence they require and deserve psychological help. Best practice will be to advocate for the quicker processing of individuals through the CJS thus avoiding the negative effects of (prolonged) incarceration. But limited resources are blamed for the lag in processing cases. With the high ratio of ATPs in proportion to convicted offenders in some (mostly developing) countries, there is a dire need for policy makers in these regions to develop alternatives to incarceration or for the sake of the wellbeing of detained individuals structure programs that will at least maintain an optimal level of wellbeing while seeking feasible alternatives to incarceration. Human rights activists and researchers could put more pressure to improve activities on the CJS in these regions.

Offending literature is rife with offence-specific treatments (e.g., anger management for violent offenders) reflecting the progress of offender management in more advanced countries. But with less developed countries having a larger proportion of non-convicted persons, for which this study provides evidence of distress and poor wellbeing, a framework addressing their needs is critically needed. Perhaps interventions for incarcerated persons could be designed to be more inclusive or specifically tailored to the needs of ATPs. As it may not be technically and morally adequate to include ATPs in offender rehabilitation programs, they could benefit from programs designed to maintain wellbeing. Studies (e.g., Evershed, 2010; Glorney et al., 2010) exploring forensic service users consider maintaining optimal wellbeing to be an essential need in secure settings. Glorney et al. (2010) equally identified that early engagement of service users in forensic settings is critical to streamlining care. Early programs could be introduced to cushion the emotional effects of incarceration which could benefit inmates who are not yet sentenced. Psychological programs as well as engagements that could help inmates stay off the cycle of depressive and anxious ruminations can help alleviate mental distress and prevent further degeneration of mental state in ATPs.

In furtherance, the accounts of the ATPs indicate that they also desire human primary goods, a stance that maps well onto a strength-based Good Lives Model (GLM, Ward, 2002) which considers individuals (especially in forensic settings) strengths as against the risk-needs-responsivity model (RNR, Andrews & Bonta, 2006, 2010) which stresses the identification and management of (re)offending risks. In short, the GLM model could form a good theoretical framework in designing programs that can be accommodating for persons who await trial.

To policy makers and “players” in the criminal justice system, the findings of this research provide evidence that prolonged incarceration without trial could severely hamper the wellbeing of individuals. As the burden of distress is largely linked to the indeterminate nature of detention in prison, ATPs, faced with uncertainty, are forced into destructive ruminations that could lead to serious psychiatric/psychological morbidity. The effect is likely to add to the economic and personnel costs of running prisons as well as stretching the resources of the prison system. Efforts should therefore be made reduce the length of time people spend in detention awaiting trial or as remand prisoners. Again, inmates showed concerns for re-engaging in pursuing personal goods as soon as they leave the prison system suggesting that they should be included in education and skill acquisition programs that could improve their chances of engagement in gainful employment upon exiting the prison system. Nonetheless care should be taken in this process as it remains to be examined whether intervention efforts on pre/awaiting-trial inmates have detrimental consequences. Branaman and Gottlieb (2013) have suggested that pre-trial therapy may have implications for victims.

### Limitations

This study, like most qualitative studies is inherently confronted with a limited generalizability of findings due to fewness and characteristics of the sample utilized. ATPs make up a dominant percentage of offenders in Nigeria (nearly 70%, Walmsley, 2012). The situation is similar for many countries in the developing world. However, to be able to sample a wider range of people would require funds which were not at the disposal of the researchers. In addition, the lived experiences of ATPs in Western, developed economies may well be different, though literature already acknowledge significant distress in some (e.g., Brinded et al., 2001 etc.). Further (transnational/cross-cultural) studies are required to establish commonalities and differences in the experiences of ATPs in different regions of the globe. We also recognize that prisons are identified as possessing different “social climates” which determine perceived safety, opportunities for personal development and the quality of the relationship among prisoners and between prisoners and prison officers (Needs, 2016; Timko & Moos, 2004). Indeed, the experiences of the participants in this study may be in excess or even an underrepresentation of conditions obtainable in other prisons.

Another limitation is the nature of the sample used in this study. Participants were purposefully selected inmates who were free from severe psychological/psychiatric distress. Individuals with significant mental distress may be argued to manifest the extreme effects of imprisonment and so their experiences would have given an enriched the data and as well the resulting interpretation of experience of imprisonment without trial. Mentally distressed inmates awaiting trial will indeed make a good case for future studies. Viktor Frankl (1984) in describing the ability to weather the tough dehumanising situations such as concentration camps, identified that few have the ability to adopt a positive outlook in dire, hopeless situations. Maybe we have selected those few and processed through their experiences and by doing so, have put aside those severely impacted by imprisonment without trial. However, severe psychopathology impairs judgement, thought processes and speech and hence will raise doubts in the quality of information derived.

The use of interviews may have yielded richer information over the adopted FGD. However limited resources and restrictions on the activities of the ATPs in accordance with the prison rules made FGDs more practical. Again the social interaction dynamics inherent in FGDs have been suggested to yield richer and deeper range of data than one-to-one interviews as well as the advantage of generating a large amount of data in a short timeframe (Raibee, 2004; Thomas, MacMillan, McColl, Hale, & Bond, 1995). Additionally, in translating the discussion transcripts from Igbo, the native language of the participating inmates to English, there is a chance of losing some meaning as originally communicated by the participants in their native language (Igbo). In addition, the presence of a prison staff may have inhibited the openness of participants in the discussion. However, we judged the proceeds of the discussion to be adequate and meaning-filled.

### Recommendations

This research highlights the trauma of imprisonment as experienced by ATPs and also featured how they put up with their prolonged incarceration. A disturbing level of uncertainty characterizes the situation of the ATPs and fuels anxiety, destructive ruminations and a cascade of mental burden. This study highlights the need for more researchers to pay closer attention to the needs and experiences of inmates awaiting trial, a group largely absent in literature. Practitioners and policy makers are challenged to be more sensitive to the needs of this largely neglected category of prisoners and campaign for alternative measures to institutionalisation, particularly for this group. Further studies could also probe similar concerns in other settings in areas where they may be in lower proportion to convicted inmates. Studies could expand to highlight adjustment after prolonged incarceration without trial, their propensity towards offending/deviant behaviours etc. Though rehabilitation programmes may sound far-fetched for ATPs, general interventions designed to alleviate the traumatic experiences of imprisonment will be beneficial. On this backdrop, we stand with contemporary researchers and practitioners (e.g., Duwe, 2016; Fazel & Baillargeon, 2011) who encourage the exploration of other possible beneficial alternatives to incarceration and additionally suggest the advancement of programmes that at least support the optimal wellbeing of ATPs.

## Appendix

Discussion Guide

Introductory Statement:

We are gathered to discuss experiences as inmates who have spent long years in prison, awaiting trial. Tell me about your experience in prison

1. In the years you have been here what has it been like?
2. When you talk about or consider how long you have stayed here, what issues come up
  - with fellow inmates
  - with staff
3. What are your (daily) worries and concerns about your stay here?
4. How are you managing to keep on
